# Pharmacogenetics of Antiplatelet Drugs

**DOI:** 10.1100/tsw.2002.153

**Published:** 2002-03-21

**Authors:** Ronan Curtin, Desmond J. Fitzgerald

**Affiliations:** Department of Clinical Pharmacology, Royal College of Surgeons in Ireland, 123 St. Stephen’s Green, Dublin 2, Ireland

**Keywords:** pharmacogenetics, Pl^A2^, GPIIb/IIIa receptor, aspirin, dipyridamole, clopidogrel, GPIIb/IIIa antagonists

## Abstract

Pharmacogenetics refers to the genetic factors that influence the response to a drug, often involving genetic variations in drug metabolizing enzymes. The pharmacogenetics of antiplatelet agents is in its infancy and largely reflects variations in drug targets or related genes. One particular gene variant, the Pl polymorphism of the glycoprotein (GP) IIb/IIIa receptor, is now emerging as a probable determinant of the response to antiplatelet agents including GPIIb/IIIa antagonists. This variant may in part explain the heterogeneity in the response to GPIIb/IIIa antagonists. The Pl genotype appears to be associated with an adverse outcome in patients treated with an oral GPIIb/IIIa antagonist and may be a factor in the observed failure of these agents in unselected populations. However, there are preliminary indications that other antiplatelet agents may have an enhanced effect in Pl subjects. Further clinical trials in particular are required to definitively characterize the pharmacogenetic effect of Pl. Other polymorphisms are also likely to contribute to the pharmacogenetics of antiplatelet agents, but these await investigation.

## INTRODUCTION

Age, concurrent medications, and renal and liver dysfunction are well-characterized factors that can affect the efficacy or toxicity of drug treatment. Inherited factors are also emerging as determinants of the pharmacokinetics and pharmacodynamics of many medications. *Pharmacogenetics* is that field of science concerned with this hereditary component of drug response. Variations in genes that encode proteins involved in drug metabolism can be important determinants of a patient's response to therapy. Genetic variations in many drug metabolizing enzymes and their phenotypes have been characterized[1]. However, genetic variation in drug transporters and drug targets, as well as genetic heterogeneity underlying the disease being treated, can also modulate response to medications.

Initially, identification of inherited differences in drug metabolism was based on observations in patients who suffered adverse effects despite being treated with standard doses of a drug[2]. However the focus of research in pharmacogenetics, triggered in part by the human genome project, has shifted to identification of single nucleotide polymorphisms (SNPs) in target genes. Drug trials or disease cohorts provide an opportunity to determine whether such SNPs modify drug response. For example, a variant of the $\beta_2$ adrenergic receptor enhances the response to β-agonist therapy in asthmatics, as the receptor fails to desensitize[3]. Variants in genes that may not be targets for drugs but are involved in the disease may also influence drug response. For example, recent evidence suggests that the angiotensin converting enzyme (ACE) DD genotype modifies the response to β-blocker therapy in heart failure[4]. Of particular importance in platelet biology and pharmacology are its surface receptors. Some of these receptors, including glycoprotein (GP) IIb/IIIa are highly polymorphic[5]. It is likely that genetic variation in these receptors is the major determinant of response to antiplatelet agents. This review will focus on the pharmacogenetics of the $Pl^{A2}$ polymorphism of GPIIIa. The potential influence of other polymorphisms on antiplatelet therapy awaits investigation.

## THE PL$^{A2}$ POLYMORPHISM

The GPIIb/IIIa receptor ($\alpha_2\beta_3$) is critical to platelet aggregation and thrombus formation (see Fig. 1). Following platelet activation by agonists such as collagen, ADP, thrombin, thromboxane $A_2$, and epinephrine, the GPIIb/IIIa receptor undergoes a conformational change that allows it to bind fibrinogen and form platelet aggregates[6]. The Platelet Antigen ($Pl^A$) polymorphism results in a leucine ($Pl^{A1}$) to proline ($Pl^{A2}$) substitution at position 33 in the extracellular portion of the IIIa subunit of GPIIb/IIIa[7]. It is responsible for most cases of post-transfusion purpura or neonatal alloimmune thrombocytopenic purpura in the Caucasian population, the amino acid substitution causing a conformational change in IIIa and exposure of novel epitopes[5]. The $Pl^{A2}$ polymorphism is common in non-Asians with about 20% heterozygous and 2% homozygous for the mutation[8].

Given the importance of the GPIIb/IIIa receptor in platelet aggregation, it was postulated that the $Pl^{A2}$ polymorphism might also affect thrombotic risk. Weiss et al.[9] in a small case-control study, showed that the $Pl^{A2}$ polymorphism was associated with a 2.8-fold (95%CI 1.2–6.4) increased risk of myocardial infarction or angina. In patients less than 60 years of age, the association appeared stronger with a relative risk (RR) of 6.2 (95%CI 1.8–22.4). More than 30 case-control studies have been published since and most have not confirmed the $Pl^{A2}$ genotype as a risk factor for coronary thrombosis. There have been two recent meta-analyses of these studies, the most recent of which suggests a weak association between the $Pl^{A2}$ polymorphism and cardiovascular disease (RR 1.10, 95%CI 1.03–1.18)[10,11]. This is not surprising, as any single variant is unlikely to contribute in a major way to the risk of coronary artery thrombosis. Indeed, there is evidence of interactions between the $Pl^{A2}$ genotype and environmental factors[12] and other gene variants[13].

Attempts to define the effect of the $Pl^{A2}$ polymorphism on platelet function have yielded apparently conflicting results. Although one study has shown an increase in fibrinogen binding in response to ADP[14], others have failed to show an increased affinity of the $Pl^{A2}$ receptor for soluble fibrinogen[15,16,17]. Vijayan et al.[16] have shown increased binding by two cell lines (Chinese Hamster Ovary and Human Embryonal Kidney 293 cells) expressing the $Pl^{A2}$ isoform of GPIIb/IIIa to immobilized but not soluble fibrinogen. However, Bennett et al.[17] did not reproduce this finding in a B-lymphocyte cell line.

$Pl^{A2}$ need not affect affinity of GPIIb/IIIa for its ligand to influence thrombosis. The variant may instead affect thrombus formation by modifying "outside-in" signaling by the receptor.

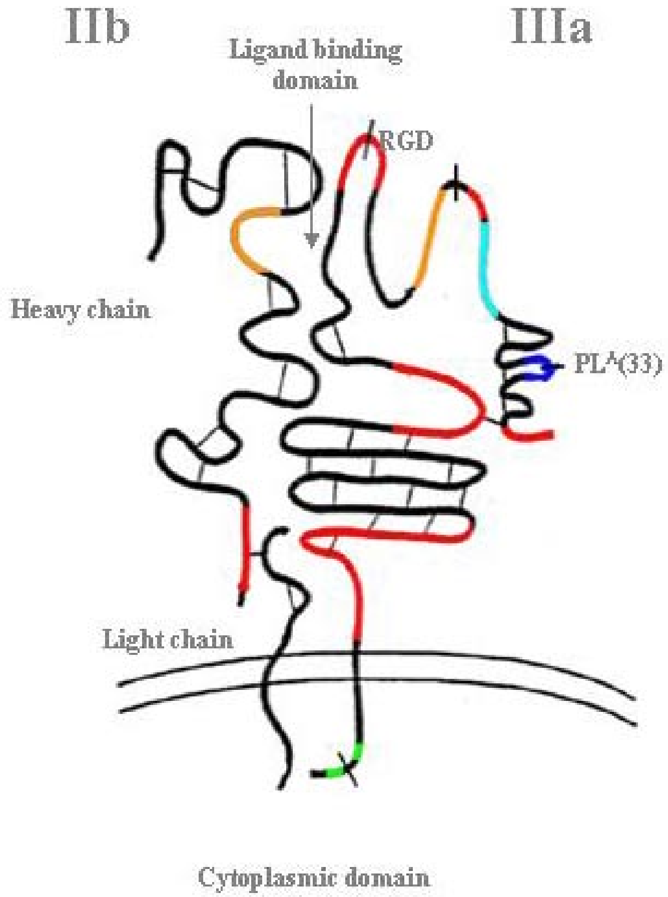

**FIGURE 1.** The GPIIb/IIIa receptor and $Pl^{A}$ polymorphism. The ligand binding site and RGD binding site are also indicated.

Outside-in signaling describes the process whereby, after ligand binding to GPIIb/IIIa, information is transmitted into the platelet that is required for aggregation[18]. Vijayan et al.[16] have shown increased peripheral F actin content, cell spreading, and fibrin clot retraction and a modest increase in $pp125^{FAK}$ phosphorylation, events characteristic of outside-in signaling, in two cell lines expressing $Pl^{A2}$ after binding to immobilized fibrinogen. Platelet α-granule release is another consequence of ligand binding to the GPIIb/IIIa receptor. Michelson et al.[19] have shown that $Pl^{A1/A2}$ and $Pl^{A2/A2}$ platelets express greater amounts of an α-granule constituent, P-selectin, in a gene-dose manner both at rest and following stimulation with a range of concentrations of ADP.

*In vitro* measurement of platelet aggregation by turbidometric assay is the standard in determining the efficacy of antiplatelet drugs. However, this method detects only larger platelet aggregates and may be relatively insensitive for detecting platelet hyperfunction[20]. Studies assessing the effect of $Pl^{A2}$ on platelet aggregation have been most inconsistent. The largest of these, a cohort of 1,422 participants in the Framingham Offspring study, concluded that $Pl^{A2}$ was associated with increased platelet reactivity as evidenced by a lower aggregation threshold concentration to epinephrine and ADP[21]. In addition, Andrioli et al.[22] found increased sensitivity of $Pl^{A2}$ platelets to thromboxane $A_2$. Although one study has reproduced the finding of increased sensitivity to epinephrine in those homozygous for $Pl^{A2}$[19], others have yielded apparently conflicting results. Lasne et al.[23] showed reduced sensitivity to ADP and TRAP in 8

and 14 $Pl^{A2}$ carriers respectively, and Bennett et al.[17] failed to show any effect of $Pl^{A2}$ on TRAP- induced aggregation over a range of concentrations of the agonist.

## ASPIRIN

Aspirin is an effective antiplatelet agent. It causes irreversible inhibition of cyclooxygenase (COX)-1 in platelets, thereby preventing formation of prostaglandin endoperoxides and thromboxane $A_2$, a platelet agonist. Aspirin has been conclusively shown to reduce the risk of further thromboembolic events in patients with a history of cardiovascular, cerebrovascular, and peripheral vascular disease[24]. Yet some patients continue to suffer thromboembolic events despite treatment with aspirin. This may reflect the fact that aspirin is a relatively weak antiplatelet agent. However it has been suggested that some patients are resistant to the antiplatelet effects of aspirin[25]. The term *aspirin nonresponder* has been used to describe those patients who show a reduced response to aspirin as measured by platelet function assays. In particular, up to 40% of patients on aspirin exhibit little or no prolongation of time to clotting in the bleeding-time test and more recently the Platelet Function Analyser (PFA-100) despite adequate suppression of platelet thromboxane $A_2$ production[26,27]. Whether these definitions of aspirin nonresponse have any impact on clinical outcome has not been established[28,29].

There is evidence to suggest that $Pl^{A2}$ may modulate response to therapy with aspirin as measured by these assays. In one study of healthy volunteers a shorter baseline bleeding time was reported in 26 $Pl^{A2}$ carriers vs. 54 $Pl^{A1A1}$ controls[30]. Following ingestion of 300 mg aspirin, the bleeding time prolonged by on average 46.5 sec in carriers vs. 112.5 sec in noncarriers, exaggerating a difference found at baseline. Bleeding time was shortened in 7 of the 26 carriers vs. 1 of the 54 controls ($p = 0.001$). The data suggest that $Pl^{A2}$ accounts in part for aspirin nonresponders.

Platelets are an important contributor to thrombin generation[31]. In addition to bleeding time, Szczeklik et al.[30] analyzed the levels of peripheral blood prothrombin fragment 1.2 (F1.2), a marker of thrombin generation, in carriers and controls before and after aspirin ingestion. Lower levels of F1.2 were found at baseline in $Pl^{A2}$ carriers vs. controls ($p = 0.02$). After treatment with aspirin, F1.2 levels showed a mild tendency to increase in carriers and decrease in controls, attenuating the baseline differences between the groups. A previous report from this group also showed reduced suppression of thrombin generation in $Pl^{A2}$ carriers on aspirin when measured at the site of vascular injury. Levels of F1.2 were similar at baseline in bleeding time blood in both groups, but after ingestion of aspirin only 9 of 15 $Pl^{A2}$ carriers vs. 23 of 25 $Pl^{A1/A1}$ subjects showed reduced thrombin generation. The relative risk of aspirin nonresponse was 6.43 (95%CI 1.38–30.02) in carriers of the $Pl^{A2}$ allele. More recently this group has further characterized the effect of $Pl^{A2}$ on thrombin generation in the bleeding-time blood of healthy volunteers before and after aspirin ingestion. By measuring prothrombin and a number of thrombin-mediated events, such as thrombin-antithrombin III production, thrombin B-chain formation, fibrinogen consumption, and factor Va generation, they showed evidence of enhanced and more rapid thrombin generation in $Pl^{A2}$ carriers. Aspirin ingestion resulted in a significant reduction in the velocity of all these processes in $Pl^{A1/A1}$ subjects but not in $Pl^{A2}$ carriers[32].

Paradoxically, three separate studies have suggested greater sensitivity to aspirin in those with the $Pl^{A2}$ polymorphism when measured by platelet aggregation *in vitro*. Cooke et al. have shown increased inhibition of epinephrine induced platelet aggregation with aspirin in a study of 11 $Pl^{A1/A2}$ subjects and 15 matched $Pl^{A1/A1}$ controls[33]. Aggregation in the absence of aspirin was similar in both groups, but there was a tenfold reduction of the $IC_{50}$ for aspirin with $Pl^{A1/A2}$ platelets ($p = 0.005$). A subsequent study from the same group confirmed increased inhibition of epinephrine induced platelet aggregation by aspirin in platelets from $Pl^{A1/A2}$ subjects, but there was a trend towards an opposite effect in platelets from $Pl^{A2/A2}$ subjects[19]. In an analysis of 17

$Pl^{A1/A1}$, 13 $Pl^{A1/A2}$, and 14 $Pl^{A2/A2}$ subjects, the $IC_{50}$ for aspirin was lower in heterozygous vs. wildtype subjects (7.4 μmol/l vs. 13.1 μmol/l, $p = 0.06$). However the $IC_{50}$ in $Pl^{A2/A2}$ subjects was slightly higher (14.0 μmol/l) than in wildtype and significantly higher than in patients heterozygous for $Pl^{A2}$ ($p = 0.015$).

The third study by Andrioli et al.[22] looked at the effect of acetylsalicylic acid on platelet aggregation in washed platelets after stimulation with arachidonic acid. Due to the small number of $Pl^{A2/A2}$ samples (n = 2) they were analyzed together with the $Pl^{A1/A2}$ samples (n = 12) and compared to $Pl^{A1/A1}$ controls (n = 49). $Pl^{A2}$ carriers were significantly more sensitive to inhibition by aspirin, with an $IC_{50}$ of 2.7 ± 0.6 μmol/l and 23.4 ± 3.3 μmol/l for $Pl^{A2}$ and $Pl^{A1/A1}$ platelets, respectively ($p < 0.005$).

These apparently conflicting results using different assays of platelet function make it difficult to deduce what effect aspirin may have on platelet inhibition in those with the $Pl^{A2}$ polymorphism. The bleeding-time test suffers from poor reproducibility and is sensitive to a host of factors not related to platelet function[34]. *In vitro* platelet aggregation is more sensitive and specific in detecting changes in platelet function; for example, it will detect changes in platelet reactivity in response to GPIIb/IIIa antagonists at much lower drug concentrations[35]. Prothrombin binds to both activated and resting GPIIb/IIIa. It has been suggested that the reduced number of occupied GPIIb/IIIa receptors resulting from enhanced inhibition of platelet aggregation by aspirin may allow greater prothrombin binding to the platelet surface and subsequent thrombin generation[36]. This theory could explain the contradictory outcome from bleeding-time tests and *in vitro* aggregation studies in $Pl^{A2}$ carriers on aspirin. However, only a prospective study with clinical endpoints will determine whether $Pl^{A2}$ has an effect on the response to aspirin.

## DIPYRIDAMOLE

Dipyridamole is an antiplatelet agent that has been shown to be particularly effective in combination with aspirin in the secondary prevention of stroke[37]. Its mechanism of action includes inhibition of phosphodiesterase and inhibition of adenosine reuptake in platelets[38]. *In vitro*, dipyridamole reduces platelet adhesion, aggregation, and platelet factor IV availability with plasma concentrations of greater than 3.5 μmol/l[39]. The plasma concentration of dipyridamole may vary as much as tenfold between individuals[40], but no predictors of this variation have been described and no pharmacogenetic studies targeting dipyridamole have been conducted.

## THE THIENOPYRIDINES

Ticlopidine and clopidogrel inhibit platelet activation by ADP by acting as antagonists of the recently cloned $P2Y_{12}$ purinergic receptor[41]. Clopidogrel is at least as effective as aspirin in reducing recurrent vascular events in high-risk patients[42]. Recently published results of the CURE study show that following an episode of unstable angina or a non-Q-wave-myocardial infarction (MI), combination therapy with aspirin and clopidogrel is superior to aspirin alone[43]. Whether all patients or only certain high-risk subgroups require combination therapy remains to be answered. Substudies from CURE may answer this question[44]. There is unpublished data indicating that some patients do not show platelet inhibition despite treatment with clopidogrel. However there are no known genetic predictors of response to clopidogrel or ticlopidine. Goldschmidt-Clermont et al.[45] studied the effect of clopidogrel in an unspecified number of $Pl^{A1/A1}$ and $Pl^{A1/A2}$ healthy volunteers and found no significant difference in platelet aggregation to a range of concentrations of epinephrine. Another potential target in pharmacogenetic studies is cytochrome P450-1A (CYP-1A), which is responsible for conversion of clopidogrel to its active metabolite[46]. Although polymorphisms have been described in this enzyme, their functional significance remains uncertain[1]. In addition, genetic variation in the drug target (the recently identified $P2Y_{12}$ receptor) could also modify the response to clopidogrel and ticlopidine.

## GLYCOPROTEIN IIB/IIIA ANTAGONISTS

The GPIIb/IIIa antagonists are effective antiplatelet agents in the setting of percutaneous coronary intervention and acute coronary syndromes when administered as intravenous infusions[47]. However, attempts to provide prolonged GPIIb/IIIa blockade with oral antagonists has met with failure and may even increase the risk of thrombotic events[48]. Abciximab, the original GPIIb/IIIa antagonist, is a chimeric murine antibody directed against GPIIb/IIIa. Subsequent intravenous and oral compounds are synthetic peptide and peptidomimetic compounds modeled on the Arg-Gly-Asp (RGD) or Lys-Gly-Asp (KGD) recognition sequences of the fibrinogen $\alpha$ and $\gamma$ chains, respectively[49].

The $Pl^{A2}$ polymorphism does not appear to alter the affinity of the receptor for small-molecule GPIIb/IIIa antagonists. The $K_i$ for RGDS has been shown to be similar in $Pl^{A1/A1}$ and $Pl^{A1/A2}$ platelets[17]. Similarly a peptidomimetic GPIIb/IIIa antagonist RWJ 53308, modeled on the KQAGD sequence of the carboxyl terminus of the fibrinogen $\gamma$ chain, shows similar binding to the receptor, regardless of $Pl^{A2}$ allotype. In addition, RWJ 53308 showed similar inhibition of TRAP-stimulated platelet aggregation at various concentrations of the antagonist in $Pl^{A1/A1}$, $Pl^{A1/A2}$, and $Pl^{A2/A2}$ platelets[17]. However, in a separate study of 23 $Pl^{A2}$ carriers (20 $Pl^{A1/A2}$, 3 $Pl^{A2/A2}$) and 24 $Pl^{A1/A1}$ controls another GPIIb/IIIa antagonist modeled on KGD, eptifibatide, appeared to be less effective in inhibiting platelet aggregation in those with the polymorphism[50]. Eptifibatide inhibited both ADP- (60 and 100 nM) and collagen- (40 nM) induced aggregation less strongly in $Pl^{A2}$ carriers with $IC_{50}$ values of 115 vs. 67 nM ($p < 0.05$) and 141 vs. 110 nM ($p < 0.05$), respectively.

On the other hand, abciximab has been reported to be more effective in inhibiting ADP-induced platelet aggregation in carriers[19]. In an analysis of 20 $Pl^{A1/A1}$, 20$Pl^{A1/A2}$, and 15$Pl^{A2/A2}$ patients, there was an enhanced inhibitory response to abciximab at therapeutic concentrations in $Pl^{A1/A2}$ compared to $Pl^{A1/A1}$ and $Pl^{A2/A2}$ patients ($p = 0.099$). The inhibitory effect was similar in $Pl^{A1/A1}$ and $Pl^{A2/A2}$ subjects. These preliminary results would appear to indicate that $Pl^{A2}$ differentially influences the response to at least some GPIIb/IIIa antagonists.

Two substudies of oral GPIIb/IIIa antagonist trials suggest that $Pl^{A2}$ may interfere with the response to these agents. In a substudy of SYMPHONY (TIMI 12), 98 patients on sibrafiban were analyzed for an effect of $Pl^{A}$ genotype on platelet aggregability and events[51]. Although there was no significant difference in platelet inhibition as measured by ADP-induced aggregation, recurrent MI occurred more commonly in the $Pl^{A2}$ group (7.4 vs. 0%, $p = 0.02$).

In OPUS, a clinical trial of the oral GPIIb/IIIa antagonist orbofiban, 10,288 patients from 29 countries were recruited within 72 h of an acute coronary syndrome and randomized to one of three groups: (1) orbofiban 50 mg twice daily for 30 days followed by 30 mg twice daily; (2) orbofiban 50 mg twice daily; or (3) placebo[52]. A subgroup of 1,014 patients, the majority of whom were from the U.S. and Canada, donated a genetic sample at a mean of 157 days. These patients were genotyped for the $Pl^{A2}$ polymorphism and its effect on response to orbofiban was examined[53]. The rate of MI was significantly higher among all $Pl^{A2}$ carriers (RR = 2.71, 95%CI 1.37–5.38, $p = 0.004$). When $Pl^{A2}$ carriers were broken down by treatment, the relative risk of a recurrent MI in those on orbofiban and placebo were 4.27 and 1.04, respectively ($p < 0.001$) (see Table 1). Of those randomized to treatment with orbofiban, $Pl^{A2}$ carriers had a more than fourfold increased risk of MI vs. noncarriers (2.46 vs. 0.59, $p = 0.08$). In addition, while there was an expected dose-dependent excess of risk of bleeding events in the noncarriers on treatment, $Pl^{A2}$ carriers showed no increased bleeding risk on treatment. The relative risk of a bleeding event on

**TABLE 1**
**Relative Risk (95% Confidence Interval) for Primary End-Point, Myocardial Infarction, and Bleeding in $Pl^{A2}$ Carriers According to Treatment**

| Event | Placebo | Treated |
|---|---|---|
| Primary endpoint[1,2] | 0.87 (0.49, 1.55) | 1.55 (1.03, 2.34)[3] |
| MI[2] | 1.04 (0.28, 3.92) | 4.27 (1.82, 10.03)[4] |
| *Bleeding*[5] | 1.22 (0.67, 2.22) | 0.59 (0.39, 0.92)[3] |

*Note*: Risks are relative to $Pl^{A2}$ noncarriers.
[1]A composite of death, MI, recurrent ischemia at rest leading to rehospitalization, or urgent revascularization or stroke.
[2]Results of a proportional hazards analysis adjusted for age and sex.
[3] $p < 0.05$.
[4] $p < 0.001$.
[5] Results of a proportional hazards analysis adjusted for age, sex, and country.

orbofiban was 1.87 (95%CI 1.29, 2.71) in $Pl^{A2}$ noncarriers vs. 0.87 (95%CI 0.46, 1.64) in $Pl^{A2}$ carriers (p =0.05). The results of this substudy indicate a reduced response to orbofiban or even a negative interaction between drug and $Pl^{A2}$ genotype. A possible explanation for these results is that $Pl^{A2}$ carriers may have a greater propensity to partial agonism when treated with GPIIb/IIIa antagonists[54]. In the setting of coronary artery disease where there is enhanced platelet activation, GPIIb/IIIa antagonists may trigger outside-in signaling and so cause further platelet activation[55]. If plasma drug levels are too low to prevent fibrinogen binding (which occurs with the oral administration of these agents), this enhanced platelet activation may ultimately lead to thrombus formation and increased clinical events.

## CONCLUSION

One single nucleotide polymorphism, the $Pl^{A2}$ variant of GPIIIa, appears to modulate the response to antiplatelet agents and GPIIb/IIIa antagonists, although studies report conflicting results. This is not surprising given the millions of single nucleotide polymorphisms in the human genome. It is very likely that many variants are involved in determining the risk of a polygenic disease such as coronary thrombosis and the response to treatment, with each variant contributing modestly to the heterogeneity in drug response. Indeed, given the number of potential variants and resulting haplotypes, it is surprising that studies have detected any effect of the $Pl^{A2}$ variant. Thus, conflicting results may reflect differences in the presence of other gene variants and gene–gene interactions, as well as environmental factors. It is also possible that $Pl^{A2}$ is in linkage with functional polymorphisms close to the GPIIIa gene and is simply a marker of another variant. Larger studies are necessary to explore this issue and to define the role of variants in other genes. Only then will it be possible to profile genes that determine the response to antithrombotic drugs and ultimately provide a means of tailoring therapy for individual patients.

## ACKNOWLEDGEMENTS

Ronan Curtin is in receipt of a European Society of Cardiology postdoctoral research fellowship in coronary thrombosis and has also received an Aventis Grant-in-Aid for Cardiovascular trainees.

## BIOSKETCH

**Dr. Ronan Curtin** is a Cardiology Research Fellow in the Department of Clinical Pharmacology, Royal College of Surgeons in Ireland. He holds MB, BCh, BAO (medical), and MSc (cardiology) degrees. His research interests include monitoring the efficacy of antiplatelet drugs as well as the impact of the $Pl^{A2}$ polymorphism of glycoprotein IIb/IIIa on platelet function and antiplatelet drug response.

**Professor Des Fitzgerald** is Chairman of the Department of Clinical Pharmacology, Royal College of Surgeons in Ireland. He holds an MD and is a Fellow of the European Society of Cardiology. His research interests include platelet adhesion receptors, the regulation of eicosanoid formation, proteomics and genomics.
